# SARS-CoV-2 Seroconversion and Pregnancy Outcomes in a Population of Pregnant Women Recruited in Milan, Italy, between April 2020 and October 2020

**DOI:** 10.3390/ijerph192416720

**Published:** 2022-12-13

**Authors:** Veronica Accurti, Bianca Gambitta, Simona Iodice, Alessandro Manenti, Simona Boito, Francesca Dapporto, Margherita Leonardi, Eleonora Molesti, Isabella Fabietti, Emanuele Montomoli, Valentina Bollati, Nicola Persico

**Affiliations:** 1 Fetal Medicine and Surgery Service, Fondazione IRCCS Ca’ Granda Ospedale Maggiore Policlinico, 20122 Milan, Italy; 2 EPIGET Lab, Department of Clinical Sciences and Community Health, University of Milan, 20122 Milan, Italy; 3 VisMederi Srl, 53100 Siena, Italy; 4 Fetal Medicine and Surgery Unit, Bambino Gesù Children’s Hospital, IRCCS, 00165 Rome, Italy; 5 Department of Molecular and Developmental Medicine, University of Siena, 53100 Siena, Italy; 6 Occupational Health Unit, Fondazione IRCCS Ca’ Granda Ospedale Maggiore Policlinico, 20122 Milan, Italy; 7 Department of Clinical Sciences and Community Health, University of Milan, 20122 Milan, Italy

**Keywords:** SARS-CoV-2, antibodies, neutralization titer, pregnancy outcomes, preterm birth hypertensive disorders, gestational diabetes, abnormal fetal growth

## Abstract

The possible link between SARS-CoV-2 infection and adverse pregnancy outcomes has so far demonstrated heterogeneous results in terms of maternal, fetal, and neonatal complications. We aim to investigate the correlation between SARS-CoV-2 seroconversion and/or neutralization titer and pregnancy outcomes. We analyzed a population of 528 pregnant women followed up from the first trimester of gestation until delivery. For each woman, we collected a first blood sample between 11 and 13 weeks of gestation and a second sample in the perinatal period (between peripartum and puerperium) to assess the presence of SARS-CoV-2 antibodies and/or microneutralization titer (MN titer). Data on pregnancy outcomes (gestational age at delivery, preterm birth before 34 weeks, hypertensive disorders, gestational diabetes, and abnormal fetal growth) were collected. We observed that serologic status per se is not associated with major pregnancy complications. On the contrary, the MN titer was associated with increased odds of gestational diabetes. Although we mainly reported asymptomatic SARS-CoV-2 infections and the absence of severe maternal and neonatal adverse outcomes, SARS-CoV-2 infection might challenge the maternal immune system and explain the moderate increase in adverse outcome odds.

## 1. Introduction

The first case of novel coronavirus-associated pneumonia was reported in Wuhan City, China [1] in December 2019 and a new coronavirus, named SARS-CoV-2, was identified as the cause of this severe respiratory illness, called Coronavirus Disease 19 (COVID-19) by the World Health Organization (WHO) on February 2020 and declared a pandemic on 11 March 2020.

In the last two decades two other human coronaviruses, SARS-CoV and MERS-CoV, have caused severe acute illness, giving rise to Severe Acute Respiratory Syndrome (SARS) and Middle East Respiratory Syndrome (MERS), respectively [2].

Due to immunological and physiological changes, pregnant women are usually more susceptible to respiratory pathogens and the development of severe respiratory symptoms [3]. However, the evidence available to date has shown that clinical manifestations of COVID-19 are similar in pregnant and non-pregnant women. Notably, in pregnant patients, the disease is more likely to be asymptomatic, while increased maternal age, high body mass index (BMI), and pre-existing comorbidities such as diabetes and hypertension are risk factors for severe COVID-19 disease [4].

Numerous studies and systematic reviews in the last two years have evaluated the correlation between SARS-CoV-2 infection and pregnancy outcomes, demonstrating heterogeneous results in terms of maternal, fetal, and neonatal complications. Data published so far has highlighted a relatively increased risk of adverse outcomes of pregnancy in patients with severe COVID-19 disease [5,6,7,8,9].

In this prospective study, we analyzed a population of pregnant women from the first trimester of gestation until delivery, to investigate the correlation between SARS-CoV-2 seroconversion and/or neutralization titer and pregnancy outcomes. The general hypothesis of the present research was that the immune response stimulated by SARS-CoV-2 infection during pregnancy might modify the pregnancy equilibrium necessary for a physiological pregnancy, leading to an alteration in maternal health and eventually influencing newborn development.

## 2. Materials and Methods

### 2.1. Subject Enrolment

To conduct the present study, we enrolled 528 pregnant women presenting at the Fetal Medicine Unit of our Hospital (Fondazione IRCCS Ca’Granda—Ospedale Maggiore Policlinico, Milan, Italy) between April 2020 and October 2020. All patients with singleton pregnancies who underwent first-trimester screening between 11 and 13 weeks of gestation were recruited.

All the participants signed a written informed consent and the study design, research aims, and measurements were approved by the Ethics Committee “Comitato Etico—Milano Area 2” of the Fondazione IRCCS Ca’ Granda Ospedale Maggiore Policlinico, 20122 Milan, Italy (approval number 357_2020), in agreement with principles of the Helsinki Declaration.

For each woman, demographic characteristics, including age, ethnicity, weight, method of conception, smoking habits, and parity were recorded. To evaluate SARS-CoV-2 immunoglobulins two blood samples were collected, the first at the time of recruitment and the second in the perinatal period (between peripartum and puerperium). Data on pregnancy outcomes (gestational age at delivery, preterm birth before 34 weeks, hypertensive disorders, gestational diabetes, and abnormal fetal growth) were collected from the hospital medical records if delivery occurred at our hospital or by telephone interview otherwise. 

All patients were interviewed on the presence of COVID-like symptoms at recruitment time, at 20–22 weeks’ gestation, and during the last blood collection. Patients were asked to report the presence of any of the following symptoms during the previous three months: fever > 37.5, cough, sore throat or cold, pneumonia or bronchitis, headache, conjunctivitis, diarrhea and/or vomiting, dyspnoea and/or tachypnoea, asthenia and/or arthralgias and/or myalgias, anosmia, ageusia, or other symptoms.

Gestational hypertension was defined as new-onset hypertension characterized by systolic blood pressure ≥140 mmHg and/or diastolic pressure ≥90 mmHg on ≥2 occasions 4 h apart following the 20th week of gestation, with no signs of proteinuria or organ failure. Preeclampsia was defined as systolic blood pressure ≥140 mmHg and/or diastolic pressure ≥90 mmHg on ≥2 occasions 4 h apart following the 20th week of gestation, accompanied by proteinuria (≥300 mg/24 h or urinary protein to creatinine ratio of ≥30 mg/mMol or two readings of at least 2+ on dipstick analysis) or by maternal organ dysfunction [10]. Gestational diabetes (GDM) was defined as any degree of glucose intolerance detected during pregnancy [11]. According to the World Health Organization (WHO, 2013) guidelines, diagnosis is defined by performing a 2 h (75 g) Oral Glucose Tolerance Test (OGTT) between the 24th and the 28th weeks of gestation. After the administration of 75 g of glucose, the presence of one or more of the following findings was considered necessary for the diagnosis of GDM: fasting plasma glucose > 5.1 mmol/L, 1-h plasma glucose > 10.0 mmol/L, or 2-h plasma glucose > 8.5 mmol/L.

Small for gestational age (SGA) and large for gestational age (LGA) neonates were defined based on a birthweight below the 5th and over the 95th percentile, respectively, according to the birthweight charts of Nicolaides et al. [12].

### 2.2. Blood Collection and Analysis

A venous blood sample (7.5 mL) was drawn in EDTA tubes, following standard procedures. Each blood sample was processed within 4 h to separate the plasma fraction. Briefly, blood EDTA was centrifuged and 1200× *g* for 15 min to obtain cell-free plasma.

### 2.3. Enzyme-Linked Immunosorbent Assay (ELISA)

Immunoglobulin (Ig) G, IgM and IgA determination in human serum samples was performed by using an in-house ELISA RBD assay [13]. 96-well ELISA plates were coated with 1µg/mL of purified recombinant Wuhan/Ancestral SARS-CoV-2 Spike-RBD protein (Arg319-Phe541) (Sino Biological, Beijing, China) expressed and purified from HEK 293 cells. Plates were incubated at 4 °C overnight and washed with 300 µL/well of Tris Buffered Saline (TBS)-0.05% Tween 20 (T-TBS), then blocked for 1 h at 37 °C with a solution of T-TBS containing 5% of Non-Fat Dry Milk (NFDM, Euroclone, Pero, Italy). Serum samples were diluted 1:100 in 5% NFDM/T-TBS. Plates were washed three times with T-TBS, then 100 μL of each sample dilution was added to the plates and incubated for 1 h at 37 °C. The plates were then washed three times and 100 µL of Goat anti-Human IgG-Fc Horse Radish Peroxidase (HRP)-conjugated antibody or IgM (μ-chain) and IgA (α-chain) diluted 1:100,000 or 1:100,000 and 1:75,000, respectively, (Bethyl Laboratories, Montgomery, AL, USA) were added each well. The plates were then incubated at 37 °C for 30 min and, after three washing steps, 100 μL/well of 3,3′, 5,5′-Tetramethylbenzidine (TMB) substrate (Bethyl Laboratories, Montgomery, AL, USA) was added and incubated in the dark at room temperature for 20 min. The reaction was stopped by adding 100 μL of hydrochloric acid solution 0.5 M (Fisher Chemical, Milan, Italy) and read within 20 min at 450 nm with a SpectraMax ELISA plate (Medical Device) reader. A cut-off value was defined as three times the average of optical density OD values from negative control wells (pool of three pre-pandemic human serum samples). Samples with ODs below the cut-off value at the lowest dilution were assigned a negative value, while samples with ODs above the cut-off value at the lowest dilution were deemed positive.

### 2.4. Micro Neutralization CPE-Based Assay

For the Micro Neutralization (MN) assay, 2-fold serial dilutions of the samples (starting dilution 1:10) were prepared in duplicate in DMEM 2% FBS and added to two different 96-well plates. The plates were then incubated for 1 h at 37 °C with a standard concentration of the virus (sample–virus ratio 1:1) [14]. Following incubation, the virus–sample mixture was then added to sub-confluent Vero E6 cells to assess whether the virus had retained its infectious capacity. After 72 h of incubation cells were inspected for signs of cytopathic effect (CPE). The highest sample dilution able to completely inhibit viral growth, in terms of CPE, was regarded as the neutralization titer. A cell-only and a virus-only control were added to each row of each plate to monitor the status of the cell monolayer and the virus itself within each plate. A negative control sample (pre-pandemic serum sample) and a positive control sample (pooled plasma high positive in terms of anti-SARS-CoV-2 immunoglobulins) were included, in duplicate, in a separate plate as a control of the assay session. 

### 2.5. Statistical Analysis

Descriptive statistics were performed on all variables. Continuous variables were expressed as mean with their standard deviation (SD), while categorical variables were expressed as frequencies with percentages.

We classified the IgG, IgM, and IgA results as high positive, positive, low positive, or negative [13], according to blood sample evaluation performed during the first trimester of pregnancy (T0) and in the peripartum (T1).

For all the women showing a positive IgG result, a microneutralization (MN) assay on plasma was also performed. For women showing a positive MN assay, neutralization capacity was defined as the highest plasma dilution that maintained the neutralizing power of the antibodies (1/10-20-40-80-160-320).

Obstetric adverse outcomes were analyzed both as a single event and combined in a composite adverse outcome, including hypertensive disorders (gestational hypertension and preeclampsia), gestational diabetes, abnormal growth (large for gestational age and small for gestational age), and delivery < 34 weeks gestation. Moreover, we investigated the frequency of newborns admitted to the Neonatal Intensive Care Unit (NICU).

We applied the chi-square test or Fisher Exact test, as appropriate, to investigate the relationship between IgG and MN results (both positivity/negativity and their change over time as categorical variables) to determine how much difference exists between observed and expected counts if there were no relationship in the population. Univariate and multivariable logistic regression models were performed to investigate the association between MN titer (both during the first trimester of pregnancy (T0) and peripartum (T1)) and the outcomes. All potential confounders were included in the multivariate model after verifying the presence of an association in a univariate model. The best model selection was based on the minimization of the Akaike information criterion and the maximization of the explained variance of the model. Multivariable models were adjusted for age and BMI. When the independent variable was MN titer at T1 we add an adjustment for the difference of MN between T0 and T1, after testing for the absence of multicollinearity through correlation inspection and calculating the variance inflation factor (VIF) statistic and the tolerance value. The estimated effects were reported as odds ratios (OR) and 95% confidence intervals (CI) associated with a unit increase in MN titer. Statistical analyses were performed with SAS software (version 9.4) and R software (version 3.6.3).

## 3. Results

Among the 528 pregnant women recruited for our study, the average maternal age was 34 years (SD = 4.5 years) and the mean body mass index (BMI) was 23 (SD = 3.9). Regarding smoking habits, 460 (87.1%) patients never smoked, 41 (7.8%) patients stopped smoking at the beginning of pregnancy, and 27 (5.1%) smoked throughout pregnancy. The mean gestational age at delivery was 39.3 weeks (SD = 2.1); 75 (14.2%) patients underwent a cesarean section, 325 (61.5%) had a vaginal birth, and 29 (5.5%) had vacuum-assisted delivery. The mean neonatal weight was 3282 g. Twelve (2.3%) newborns were hospitalized in the neonatal intensive care unit (Table 1, Supplementary Table S1).

A total of 147 (27.8%) patients experienced one or more adverse pregnancy outcomes, for a total of 171 events. Abnormal growth represented the most common adverse outcome and was experienced by 46 (8.7%) women, which includes 30 (5.7%) SGA newborns and 16 (3.0%) LGA newborns. Only 5 (0.95%) patients delivered before 34 weeks. We identified 34 (6.4%) women with gestational diabetes and 11 (2.1%) with a hypertensive disorder, including 8 (1.5%) women with preeclampsia and 3 (0.6%) with gestational hypertension. We combined in a single composite adverse outcome the 171 pregnancy complications, including hypertensive disorders (gestational hypertension and preeclampsia), gestational diabetes, abnormal growth (large for gestational age and small for gestational age), and delivery < 34, as shown in Table 2.

The population symptoms are reported in Supplementary Figure S1, which highlights the prevalence of asymptomatic status in each of the three assessments.

As shown in Table 3, during the first trimester of pregnancy, 92.4% of the women showed negative IgG, while 7.6% were positive (0.8% high positive, 4.2% positive, and 2.7% low positive). We observed the presence of neutralizing anti-SARS-CoV-2 antibodies in 27 (67.5%) of the 40 women who tested positive for IgG antibodies. Ninety-seven women close to the pregnancy due date (T1) tested positive for IgG antibodies (18.4%). Among them, 1.7% were high positive, 9.7% positive, 7% low positive, and 79 (81.4%) showed neutralizing antibodies.

Venn diagrams showing the number of women testing positive for IgM, IgG, IgA, and/or MN assay on plasma during the first trimester and peripartum are shown in Supplementary Figures S2 and S3.

Changes in immunoglobulin levels between T0 and T1 for IgM, IgG, IgA, and MN are reported in Table 4. We observed 64 women (12.1%) developing IgG between the first trimester and the peripartum, suggestive of an infection experienced during pregnancy.

In Supplementary Figure S4 we present the histogram showing changes in IgG, IgM, IgA antibodies, and MN over time (T0 and T1), and all the possible combinations.

We further evaluated the IgG and MN assay results in association with maternal and neonatal outcomes, evaluated as a single obstetric complication or a composite adverse outcome score. Unadjusted results of chi-square tests for association to evaluate differences in adverse outcomes in women’s IgG status at either T0 or T1, and according to IgG change over time, are reported in Table 5 and Supplementary Table S2, respectively. LGA newborns were more frequent in pregnancies positive for IgG at any time (6.7% vs. 2.1%; *p* = 0.023). No differences in adverse outcomes were observed in women with positive MN (Supplementary Tables S3 and S4).

Increasing MN titer during the first trimester of pregnancy was associated with 6.3% higher odds of gestational diabetes (*p* = 0.010). Moreover, the MN titer close to the pregnancy due date was associated with a 4.2% increase in the composite adverse outcome (OR = 1.04, 95% CI 1.0–1.08) and an 8% increase in the odds of gestational diabetes (OR = 1.08, 95% CI 1.02–1.14) (Table 6). Other variables (i.e., parity, ethnicity, smoking habits, and gestational age) were tested as confounder variables potentially associated both with antibody positivity and with the adverse outcomes, but were found not to be associated.

The increased odds of gestational diabetes associated with MN titer at T0 was confirmed, by multivariate analysis, after age and BMI adjustment and raised to 10% at T1. (Table 7).

Interestingly, although we observed increased differences in MN titer between the first and second withdrawals, the odds of gestational diabetes increased by 8.2%, irrespective of the MN value at T1, age, and BMI (OR = 1.08, 95% CI 1.02–1.15). Lastly, we observed 5% higher odds of NICU hospitalizations associated with increasing MN titer both during the first trimester of pregnancy (*p* = 0.012) and close to the pregnancy due date (*p* = 0.015).

## 4. Discussion

In the present study, we observed that serologic status per se is not associated with major pregnancy complications.

In contrast, the level of neutralizing antibodies (MN titer), a parameter that has been associated with the protection from the COVID-19 disease [15] but also with COVID-19 symptoms’ severity [16], has been associated with some adverse outcomes. Particularly of interest is the relationship between the MN titer and the odds of developing gestational diabetes.

This study focused on a population of pregnant women in an area with a high prevalence of SARS-CoV-2, in a period in which COVID vaccination was not yet available. Moreover, the study population underwent blood sampling both at the beginning of pregnancy and near term. This enabled us to detect seroconversion during pregnancy and to determine a possible correlation with adverse maternal and fetal outcomes.

Studies conducted in Europe and America during 2020 showed a SARS-CoV-2 seroprevalence in pregnancy between 4.7% and 16.1%. The serological evaluation was mainly carried out at the time of hospitalization, at the time of delivery, or randomly during pregnancy. However, to our knowledge, this is the first study assessing maternal and neonatal outcomes in a population sampled both in the first trimester and in the perinatal period [17,18,19,20,21,22,23,24,25].

Previous studies have reported on the rate of seroconversion for SARS-CoV-2 during the three trimesters of pregnancy [26]. Staszewski et al. analyzed SARS-CoV-2 IgG in 149 pregnant women in the first and second trimesters and at the time of delivery. They observed an antibody response throughout pregnancy, finding seropositivity between 12.1% and 16.1% [26], which is comparable to what we observed (seroprevalence: 19.7%, seroconversion: 12.1%) However, none of these studies analyzed the correlation between seroprevalence and clinical outcomes.

We did not find any correlation between the presence of SARS-CoV-2 IgG antibodies or the ability to inhibit the virus and the development of preeclampsia. This result is consistent with previous studies [26,27,28,29,30,31]. However, Jemieson et al. demonstrated a correlation between preeclampsia and severe COVID disease [32], while in a retrospective cohort study Ko et al. observed a link between preeclampsia and any manifestation of SARS-CoV-2 infection [33]. These results were confirmed by Conde-Agudelo and Romero [34]. Despite the limited number of seropositive pregnant women, the data in our study allowed us to confirm good maternal, fetal, and neonatal outcomes when the disease is asymptomatic or mild.

Furthermore, our data were consistent with the vast majority of current studies which point out that there is no association between mild or asymptomatic COVID-19 and gestational diabetes (GDM) [21,27,28,35,36,37,38]. Conversely, Adhikari and Jamienson reported a correlation between GDM and severe COVID-19 disease [2,18]. Our findings failed to demonstrate an association between SARS-CoV-2 IgG seropositivity and GDM; however, our data showed a correlation between an increased odds of developing GDM and the antibodies’ ability to neutralize the virus: the greater the microneutralization assay titer, the greater the odds of this complication of pregnancy.

This particular association could be due to the immunological response rather than to the virus infection or COVID-19 disease, highlighting the already-known association between inflammatory processes and pregnancy complications [39].

In our cohort only 0.9% of patients gave birth before 34 weeks of gestation, with a mean gestational age at delivery of 39 weeks and a mean neonatal weight of 3282 g, demonstrating no correlation with SARS-CoV-2 IgG seropositivity and microneutralization titer.

Our results on preterm birth were consistent with data from several studies [5,27,29,40,41,42,43,44], although others report an increased risk of preterm delivery among pregnant women with SARS-COV-2 infection [4,32,33,37,45,46,47,48,49,50,51]. However, Khalil et al. suggest that, during COVID-19 disease, iatrogenic preterm delivery is increased rather than spontaneous [52].

The contrasting results reflect the heterogeneity of studied populations. Adhikari, Crovetto, and Overtoom did not find an increased risk of preterm birth among pregnant patients affected by COVID-19. However, the first two reported this complication in cases of severe disease [28,30] or, as Overtoom considered [53], when there is a symptomatic form of the disease at the time of delivery. Also, the WAPM Group on COVID-19 detected a high incidence of preterm birth among symptomatic pregnant women [54]. According to the data published so far, in our mainly asymptomatic population, the rate of preterm delivery was low, despite the presence of SARS-CoV-2 antibodies.

Regarding birth weight, the findings from this study were consistent with previous data. Son and Llorca demonstrated no significant differences between pregnant women tested and classified as negative for SARS-CoV-2 and positive patients in terms of small-for-gestational-age (SGA) or large-for-gestational-age (LGA) fetuses [29,41]. Some studies highlighted the association between low birth weight and COVID-19 disease [46,55], but, as suggested by Han [46], this could be related to prematurity. Also, Crovetto et al. confirmed differences between symptomatic and asymptomatic infection with a severe increase of SGA newborns in symptomatic COVID-19 women [28].

The main strength of our study was that a microneutralization assay was applied to all the positive IgG samples to assess the ability of the antibodies to neutralize SARS-CoV-2 in vitro. Virus neutralization assays measure antibodies’ ability to counterbalance viral growth during in vitro tests and it is considered the gold standard for measuring antibody activity and excluding false-positive results.

On the contrary, the main limitation of the present study is that the number of women developing gestational diabetes, or any other adverse pregnancy outcome, was small, thus making this study explorative.

Another possible limitation of our study is a potential bias due to unmeasured confounding: e.g., information on GDM that occurred in previous pregnancies, for multiparous women, might be a risk factor for GDM in the current pregnancy, but this information was not collected for the present group of women.

## 5. Conclusions

In summary, our findings reported information about maternal and neonatal outcomes of a non-vaccinated pregnant population in an area that was heavily affected by the pandemic, showing mainly asymptomatic SARS-CoV-2 infections and the absence of severe adverse outcomes. Although the study design of the present investigation did not allow us to confirm that the relationship was causal, the possible correlation between immune response and gestational diabetes could be explained by the direct effect of the novel coronavirus on the host’s immune system. However, additional studies on larger populations of seroconverted pregnant women are needed.

## Figures and Tables

**Table 1:** Demographics and obstetric characteristics of 528 pregnant women and neonatal parameters.

Characteristic	Mean ± SD or Frequency (%)
Age, year	34.0 ± 4.5
Gestational age at sample, weeks	11.7 ± 0.8
**Anthropometric features**	
Maternal weight, Kg	62.5 ± 11.3
BMI, Kg/m^2^	23.0 ± 3.9
*Categorical BMI*	
Underweight (BMI < 18.5)	27 (5.1%)
Lean (18.5 ≤ BMI < 25)	376 (71.2%)
Overweight (BMI ≥ 25)	125 (23.7%)
*Ethnicity*	
White (European, Middle Eastern, North African, Latin American)	509 (96.4%)
East Asian (Chinese, Korean, Japanese)	5 (0.9%)
Black (African, Caribbean, African-American)	4 (0.8%)
South Asian (Indian, Pakistani, Bengali)	9 (1.7%)
Other	1 (0.2%)
**Smoking habits, *n* (%)**	
Never smoked	460 (87.1%)
Stopped during pregnancy	41 (7.8%)
Smoker	27 (5.1%)
**Obstetric History**	
*Parity*	
Nulliparity	326 (61.7%)
Multiparity	202 (38.3%)
**Delivery**	
Gestational age at delivery, weeks	39.3 ± 2.1
Caesarean section	174 (33.0%)
Vaginal	325 (61.5%)
Vacuum	29 (5.5%)
**Neonatal parameters**	
Weight, g	3281.5 ± 456.6
*Sex*	
Female	260 (49.2%)
Male	268 (50.8%)
NICU admission	12 (2.3%)

**Table 2:** Obstetric complications.

	Frequency (%)
Hypertensive disorders	11 (2.1%)
*Gestational hypertension*	*3 (0.6%)*
*Preeclampsia*	*8 (1.5%)*
Gestational diabetes	34 (6.4%)
Abnormal growth	46 (8.7%)
*Large for gestational age*	*16 (3.0%)*
*Small for gestational age*	*30 (5.7%)*
Delivery < 34 weeks	5 (0.95%)
Emergency cesarean section	75 (14.2%)

**Table 3:** IgG, Microneutralization assay titers, IgM, and IgA in the first trimester of pregnancy (T0) and peripartum (T1).

Test (*n* = 528)	First Trimester (T0)	Peripartum (T1)
	*n* (%)	*n* (%)
**IgG antibodies**		
Negative	488 (92.4%)	431 (81.6%)
Positive	40 (7.6%)	97 (18.4%)
*High Positive*	*4 (0.8%)*	*9 (1.7%)*
*Positive*	*22 (4.2%)*	*51 (9.7%)*
*Low Positive*	*14 (2.7%)*	*37 (7%)*
Microneutralization (*n* = 137)	27 (67.5%)	79 (81.4%)
*Microneutralization assay titers*		
*10*	*13 (48.1%)*	*28 (35.4%)*
*20*	*8 (29.6%)*	*22 (27.8%)*
*40*	*5 (18.5%)*	*16 (20.3%)*
*80*	*1 (3.7%)*	*9 (11.4%)*
*160*	*0 (0%)*	*2 (2.5%)*
*320*	*0 (0%)*	*2 (2.5%)*
**IgM antibodies**		
Negative	516 (97.7%)	476 (90.2%)
Positive	12 (2.3%)	52 (9.8%)
*High Positive*	*0 (0%)*	*1 (0.2%)*
*Positive*	*3 (0.6%)*	*20 (3.8%)*
*Low Positive*	*9 (1.7%)*	*31 (5.9%)*
**IgA antibodies**		
Negative	525 (99.4%)	505 (95.6%)
Positive	3 (0.6%)	
*High Positive*	0 (0%)	1 (0.2%)
*Positive*	0 (0%)	5 (1.0%)
*Low Positive*	3 (0.6%)	17 (3.2%)

**Table 4:** Change in IgG, IgM, IgA and MN between the first trimester of pregnancy (T0) and peripartum (T1).

Tests Changes over Time	*n* (%)
IgG change	
Unchanged Negative	424 (80.3%)
Negativized	7 (1.3%)
Positivized	64 (12.1%)
Unchanged Positive	33 (6.3%)
MN change *	
Unchanged Negative	51 (83.7%)
Negativized	7 (1.3%)
Positivized	59 (11.2%)
Unchanged Positive	20 (3.8%)
IgM change	
Unchanged Negative	470 (89.0%)
Negativized	6 (1.1%)
Positivized	46 (8.7%)
Unchanged Positive	6 (1.1%)
IgA change	
Unchanged Negative	504 (95.5%)
Negativized	1 (0.2%)
Positivized	21 (4.0%)
Unchanged Positive	2 (0.4%)

* MN change was evaluated among 104 women with any IgG positivity at T0 or T1.

**Table 5:** Adverse outcomes in women with any positivity to IgG at T0 or T1.

	IgG		*p*-Value
	Positive	Negative	
Adverse outcome	18 (17.3%)	68 (16.0%)	0.753
Hypertensive disorders	3 (2.9%)	8 (1.9%)	0.460
*Gestational hypertension*	0 (0%)	3 (0.7%)	1.000
*Preeclampsia*	3 (2.9%)	5 (1.2%)	0.195
Gestational diabetes	6 (5.8%)	28 (6.6%)	0.756
Abnormal growth	9 (8.7%)	37 (8.7%)	0.981
*Large for gestational age*	*7 (6.7%)*	*9 (2.1%)*	*0.023*
*Small for gestational age*	*2 (1.9%)*	*28 (6.6%)*	*0.065*
Delivery < 34 weeks	1(1.0%)	4 (0.9%)	1.000

**Table 6:** Association of adverse outcomes with MN titer measured the first trimester of pregnancy (T0) and peripartum (T1).

Independent Variable	Outcomes	OR	95% LCI	95% UCI	*p*-Value
MN at T0 ^a^					
	Composite adverse outcome	1.035	0.999	1.072	0.057
	Hypertensive disorders	1.007	0.928	1.093	0.866
	Gestational diabetes	1.063	1.015	1.112	0.010
	Abnormal growth	1.008	0.959	1.058	0.763
	Delivery < 34 weeks	1.273	0.040	40.073	0.891
MN at T1 ^b^					
	Composite adverse outcome	1.042	1.003	1.082	0.036
	Hypertensive disorders	1.007	0.926	1.096	0.864
	Gestational diabetes	1.080	1.022	1.142	0.007
	Abnormal growth	1.001	0.946	1.059	0.788
	Delivery < 34 weeks	1.492	0.040	55.61	0.828

^a^ Univariate logistic models; ^b^ logistic models adjusted for the difference in MN titer between T1 and T0; LCI, lower confidence interval; UCI, upper confidence interval.

**Table 7:** Odds ratios (OR) of adverse outcomes, gestational diabetes, or need for NICU in association with MN titer at the first trimester of pregnancy (T0) and at peripartum (T1) after age and BMI adjustment.

Independent Variables	Adverse Outcomes		Gestational Diabetes		NICU	
	OR (95% CI)	*p*-Value	OR (95% CI)	*p*-Value	OR (95% CI)	*p*-Value
MN titer at T0	1.032 (0.994–1.070)	0.097	1.070 (1.014–1.130)	0.014	1.053 (1.012–1.097)	0.012
Age	1.044 (0.936–1.165)	0.440	1.198 (1.001–1.435)	0.049	0.908 (0.798–1.033)	0.144
BMI	1.058 (0.946–1.183)	0.321	1.042 (0.862–1.259)	0.673	1.011 (0.883–1.158)	0.871
MN titer at T1	1.039 (0.998–1.082)	0.062	1.096 (1.024–1.173)	0.008	1.052 (1.010–1.097)	0.015
MN change between T0 and T1	1.034 (0.996–1.074)	0.084	1.082 (1.016–1.152)	0.014	1.002 (0.975–1.030)	0.871
Age	1.048 (0.939–1.170)	0.404	1.216 (1.011–1.462)	0.038	0.907 (0.797–1.033)	0.142
BMI	1.045 (0.931–1.172)	0.457	1.010 (0.800–1.274)	0.936	1.013 (0.884–1.162)	0.851

NICU, newborns experiencing the neonatal intensive care unit; CI, confidence interval; BMI, body mass index.

## Data Availability

Not applicable.

## Supplementary Materials

The following supporting information can be downloaded at: https://www.mdpi.com/article/10.3390/ijerph192416720/s1, Table S1: Characteristics of 12 newborns admitted to the Neonatal Intensive Cure Unit (NICU); Table S2: Adverse outcomes according to IgG change over time.; Table S3: Adverse outcomes in women with any MN positivity; Table S4: Adverse outcomes according to MN change over time. Figure S1: Symptoms over the study period. Patient were investigated at recruitment (blu bar), halfway through the study (red bar) and during the last blood sampling close to delivery (green bar).; Figure S2: Venn diagram showing IgG, IgM, IgA, and microneutralization assay titre in the first trimester of pregnancy (T0; left panel) and peripartum (T1; right panel)*;* Figure S3: Venn diagram showing the number of subjects testing positive for IgG, IgM, IgA antibodies, or MN during first trimester of pregnancy (T0) and peripartum (T1).; Figure S4: Histogram of all the possible combinations of positivity/negativity of IgG, IgM, IgA antibodies, and MN, at T0 and T1.
