# Supplementary material for: SARS-CoV-2 Seroconversion and Pregnancy Outcomes in a Population of Pregnant Women Recruited in Milan, Italy, between April 2020 and October 2020
Source: Int J Environ Res Public Health. 2022 Dec 13;19(24):16720. doi: 10.3390/ijerph192416720 (PMC9778651; doi:10.3390/ijerph192416720)
Supplement: Supplementary file 1 [file ijerph-19-16720-s001.zip › ijerph-1970750-supplementary.pdf]

Supplementary Table S1. Characteristics of 12 newborns admitted to the Neonatal Intensive Cure Unit (NICU).

Neonate	IgG T0 mother	IgG T1 mother	Gestational age at delivery	Mode of delivery	Days in NICU	CPAP	RDS	IVH	Notes
1	Neg	Neg	26.4	CS	96	No	Yes	Yes	Sepsis
2	Neg	Neg	32.7	Vaginal	38	No	No	No	Prematurity
3	Neg	Neg	41.6	CS	14	Yes	No	No	Perinatal infection
4	Neg	Neg	38.6	CS	23	No	No	No	Urinary tract malformation
5	Pos	Pos	33.9	CS	105	Yes	No	No	Placenta previa
6	Neg	Neg	38.9	CS	42	No	No	No	Prader Willi Sd
7	Neg	Neg	36.1	Vaginal	25	Yes	No	No	Anemia: fetomaternal hemorrage
8	Neg	Neg	41.6	Vacuum	13	No	No	No	Perinatal Infection
9	Neg	Neg	37.6	CS	11	No	No	No	Feeding difficulties
10	Neg	Neg	38.4	Vaginal	13	No	No	No	Arrhythmia
11	Pos	Neg	38.9	CS	111	No	Yes	No	Left Congenital diaphragmatic hernia
12	Pos	Pos	39.9	CS	4	No	No	No	Meconium aspiration syndrome

CPAP: Continuous airway positive pressure; RDS: respiratory distress syndrome; IVH: Intraventricular hemorrhage.

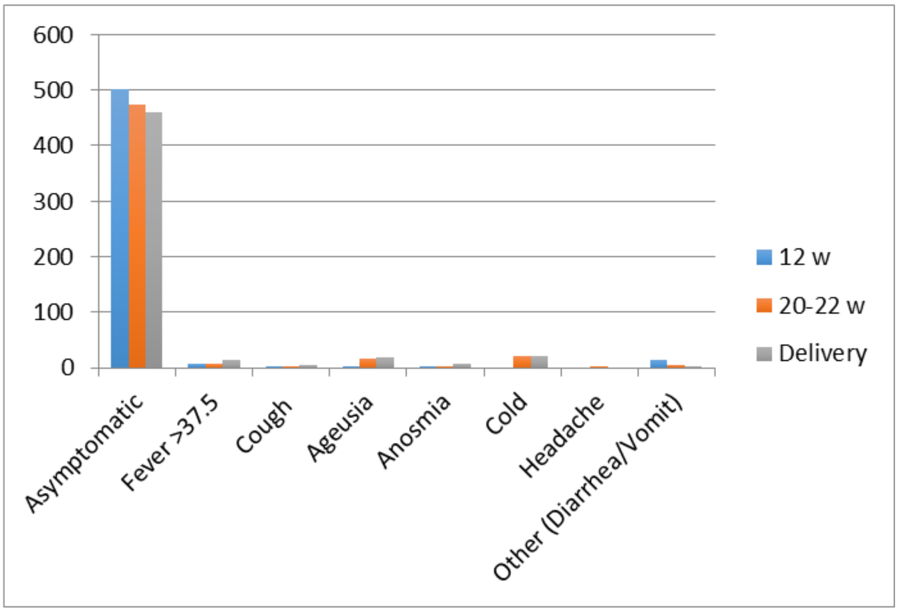

Supplementary Figure S1. Symptoms over the study period. Patient were investigated at recruitment (blu bar), halfway through the study (red bar) and during the last blood sampling close to delivery (green bar).

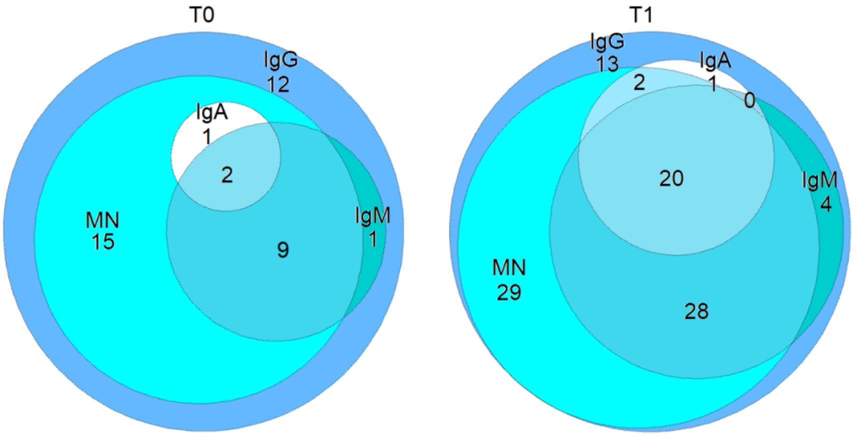

Supplementary Figure S2. Venn diagram showing IgG, IgM, IgA, and microneutralization assay titre in the first trimester of pregnancy (T0; left panel) and peripartum (T1; right panel).

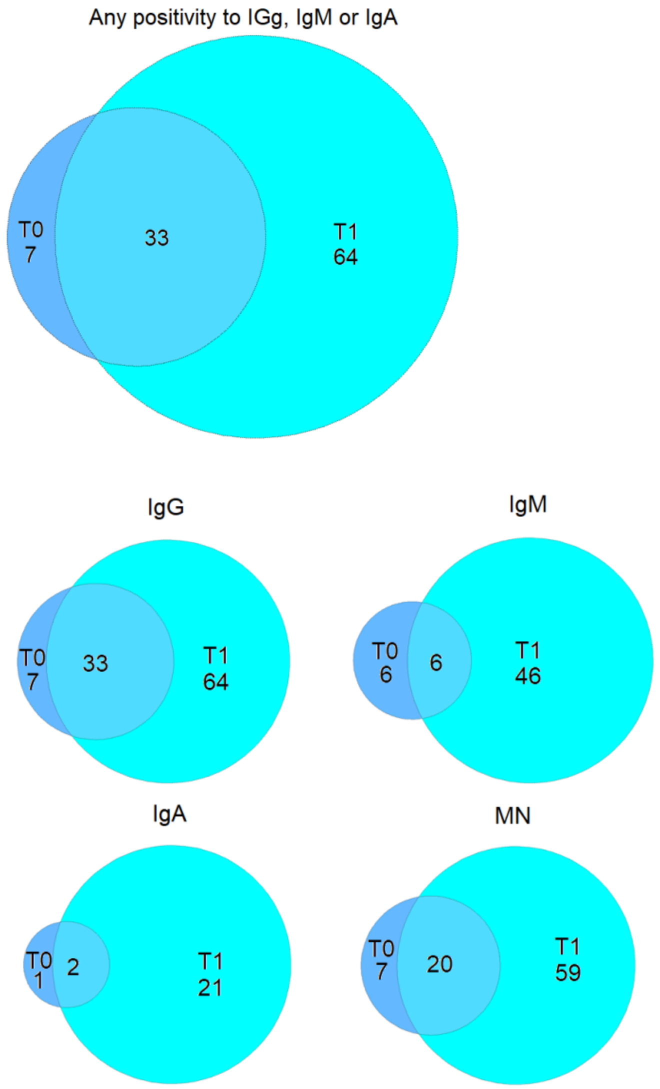

Supplementary Figure S3. Venn diagram showing the number of subjects testing positive for IgG, IgM, IgA antibodies, or MN during first trimester of pregnancy (T0) and peripartum (T1).

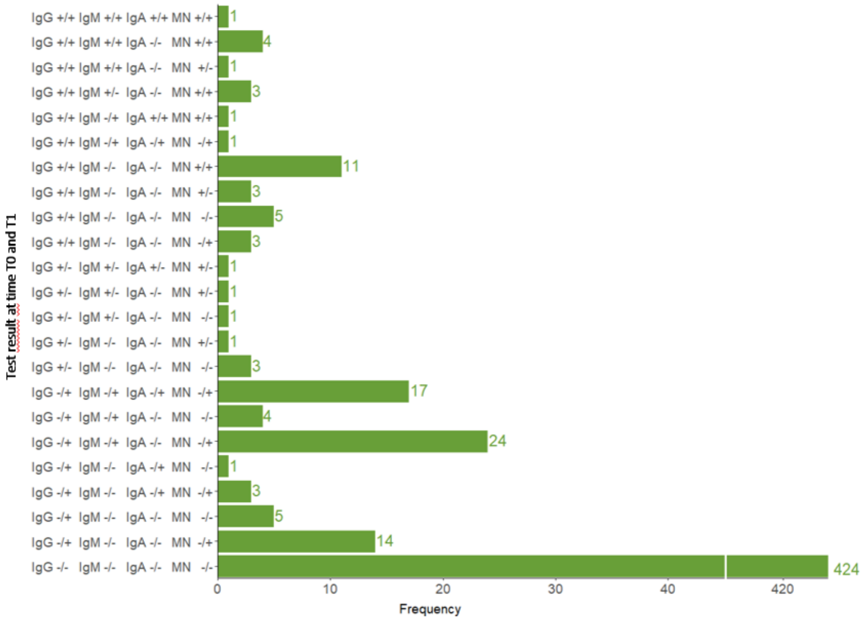

Supplementary Figure S4. Histogram of all the possible combinations of positivity/negativity of IgG, IgM, IgA antibodies, and MN, at T0 and T1.

Supplementary Table S2. Adverse outcomes according to IgG change over time.

	**IGG change**				**p-value**
	**Negativized**	**Positivized**	**Unchanged Negative**	**Unchanged Positive**	
	**+/-**	**-/+**	**-/-**	**+/+**	
	**n=7**	**n=64**	**n=424**	**n=33**	
Adverse outcome	0 (0%)	10 (15.6%)	68 (16.0%)	8 (24.2%)	0.402
Hypertensive disorders	0 (0%)	1 (1.6%)	8 (19%)	2 (6.1%)	0.327
Gestational hypertension	*0 (0%)*	*0 (0%)*	*3 (0.7%)*	*0 (0%)*	*1.000*
Preeclampsia	*0 (0%)*	*1 (1.6%)*	*5 (1.2%)*	*2 (6.1%)*	*0.171*
Gestational diabetes	0 (0%)	3 (4.7%)	28 (6.6%)	3 (9.1%)	0.777
Abnormal growth	0 (0%)	6 (9.4%)	37 (8.7%)	3 (9.1%)	0.976
Large for gestational age	*0 (0%)*	*5 (7.8%)*	*9 (2.1%)*	*2 (6.1%)*	*0.060*
Small for gestational age	*0 (0%)*	*1 (1.6%)*	*28 (6.6%)*	*1 (3.0%)*	*0.419*
Delivery < 34 weeks	0 (0%)	0 (0%)	4 (0.9%)	1 (3.0%)	0.414

Supplementary Table S3. Adverse outcomes in women with any MN positivity.

	MN		p-value
	**Positive n=86**	**Negative n=442**	
Adverse outcome	15 (17.4%)	71 (16.1%)	0.750
Hypertensive disorders	2 (2.3%)	9 (2.0%)	0.697
Gestational hypertension	*0 (0%)*	*3 (0.7%)*	*1.0*
Preeclampsia	*2 (2.3%)*	*6 (1.4%)*	*0.663*
Gestational diabetes	6 (7.0%)	28 (6.3%)	0.824
Abnormal growth	7 (8.1%)	39 (8.8%)	0.837
Large for gestational age	*5 (5.8%)*	*11 (2.5%)*	*0.157*
Small for gestational age	*2 (2.3%)*	*28 (6.3%)*	*0.202*
Delivery < 34 weeks	1 (1.2%)	4 (0.9%)	0.590

Supplementary Table S4. Adverse outcomes according to MN change over time.

	MN change				p-value
	**Negativized +/-**	**Positivized -/+**	**Unchanged Negative -/-**	**Unchanged Positive +/+**	
	**n=7**	**n=59**	**n=51**	**n=20**	
Adverse outcome	1 (14.3%)	10 (17.0%)	71 (16.1%)	4 (20%)	0.912
Hypertensive disorders	0 (0%)	1 (1.7%)	9 (2.0%)	1 (5.0%)	0.514
Gestational hypertension	*0 (0%)*	*0 (0%)*	*3 (0.7%)*	*0 (0%)*	*1.000*
Preeclampsia	*0 (0%)*	*1 (1.7%)*	*6 (1.4%)*	*1 (5.0%)*	*0.294*
Gestational diabetes	1 (14.3%)	3 (5.1%)	28 (6.3%)	2 (10.0%)	0.440
Abnormal growth	0 (0%)	5 (8.5%)	39 (8.8%)	2 (10.0%)	0.969
Large for gestational age	*0 (0%)*	*4 (6.8%)*	*11 (2.5%)*	*1 (5.0%)*	*0.202*
Small for gestational age	*0 (0%)*	*1 (1.7%)*	*28 (6.3%)*	*1 (5.0%)*	*0.537*
Delivery < 34 weeks	0 (0%)	1 (1.7%)	4 (0.9%)	0 (0%)	0.590
